# Impact of renin-angiotensin system inhibitors and beta-blockers on dental implant stability

**DOI:** 10.1186/s40729-021-00309-y

**Published:** 2021-04-08

**Authors:** Babak Saravi, Andreas Vollmer, Gernot Lang, Nicholai Adolphs, Zhen Li, Verena Giers, Peter Stoll

**Affiliations:** 1 Department of Orthopedics and Trauma Surgery, Medical Centre-Albert-Ludwigs-University of Freiburg, Faculty of Medicine, Albert-Ludwigs-University of Freiburg, Hugstetterstrasse 55, 79106 Freiburg, Germany; 2 Prof. Dr. Dr. Stoll & Partner Wilhelmstraße 3, 79098 Freiburg, Germany; 3 AO Foundation, Davos, Switzerland

**Keywords:** Implant stability, ISQ, Antihypertensive, Renin-angiotensin system, Beta-blockers, Implant, Osseointegration, Bone remodeling

## Abstract

**Background:**

Current experimental research suggests antihypertensive medication reduces the failure risk of dental implants due to enhanced bone remodeling. However, evidence from clinical studies evaluating the impact of antihypertensive medication on implant stability is lacking.

**Methods:**

We retrospectively analyzed 377 implants in 196 patients (46 implants inserted in antihypertensive drug users (AH) and 331 implants in non-users (NAH)) for implant stability measured by radiofrequency analysis, and we determined the implant stability quotient (ISQ). AH subgroups were stratified by the use of beta-blockers, renin-angiotensin system (RAS) inhibitors, and both of the aforementioned. The impact of antihypertensive medication on ISQ values at implant insertion (primary stability) and implant exposure (secondary stability) was analyzed by a linear regression model with a regression coefficient and its 95% confidence interval (95% CI), adjusted for potential confounders.

**Results:**

Time between implant insertion and implant exposure was 117.1 ± 56.6 days. ISQ values at insertion were 71.8 ± 8.7 for NAH and 74.1 ± 5.6 for AH, respectively. ISQ at exposure was 73.7 ± 8.1 for NAH and 75.7 ± 5.9 for AH. Regression analysis revealed that none of the AH subgroups were significantly related to ISQ at implant insertion. However, renin-angiotensin system inhibitors (RAS) were significantly associated with higher ISQ values at exposure (reg. coeff. 3.59, 95% CI 0.46–6.71 (*p*=0.025)).

**Conclusions:**

Outcome of the present study indicates enhanced bone remodeling and osseointegration following dental implant insertion in patients taking RAS inhibitors than in non-users. Future randomized prospective studies must confirm these indicative results.

## Background

Hypertension is a leading risk factor for cardiovascular diseases and mortality [1–3]. It has been estimated that 1.31 billion adults (31.1%) worldwide suffered from hypertension in 2010 and that this number will increase to 1.56 billion in 2025 [1, 3, 4]. Clinical studies provide evidence that lower blood pressure reduces the risk of cardiovascular complications [5]. Antihypertensive medication, including inhibitors of the renin-angiotensin system (RAS) and beta-blockers (BB) are frequently prescribed drugs. Especially the polytherapy with calcium channel blocker, BB, or RAS inhibitors have significantly increased among adults with hypertension in the last years [6, 7]. The prevalence of hypertension is correlated with age and is reported to be over 66% in the older population (>60 years) [8]. As the number of missing teeth is also correlated with age, this patient cohort is regularly seen by dental implant surgeons and dentists [9].

In recent years, several studies indicated a link between hypertension and periodontal tissue metabolism and diseases [10, 11]. Especially the periodontal tissue renin-angiotensin system (tRAS) has been suggested to be involved in the progression of inflammation and bone loss mediated by angiotensin II (ATII), the main effector of the RAS [12, 13]. Recent preclinical studies provided evidence that RAS-inhibitors reduce periodontal inflammation and increase alveolar bone volume [12, 14–16]. As the success of osseointegrated implants highly depends on bone formation and remodeling processes, there might be a benefit for antihypertensive drug users who were treated with dental implants. Angiotensin II type 1 receptor blockers (ARB) and angiotensin-converting enzyme (ACE) inhibitors have shown to (1) inhibit the release of mediators that activate osteoclasts via angiotensin II type 1 receptors on osteoblasts, (2) increase blood flow in bone marrow capillaries, increase free Ca2+ ion levels in plasma while decreasing parathormone, and overall (3) prevent harmful effects of ATII on bones, especially the osteoclast activation with ATII [17–20]. However, data from clinical studies are scarce. Few studies investigated the impact of other antihypertensive drug groups on bone-relevant parameters suggesting higher bone mineral density and reduced bone fracture risk in patients treated with beta-blockers compared to calcium channel blockers, thiazides, or patients who never used antihypertensive medication [21–24].

**Fig. 1:**
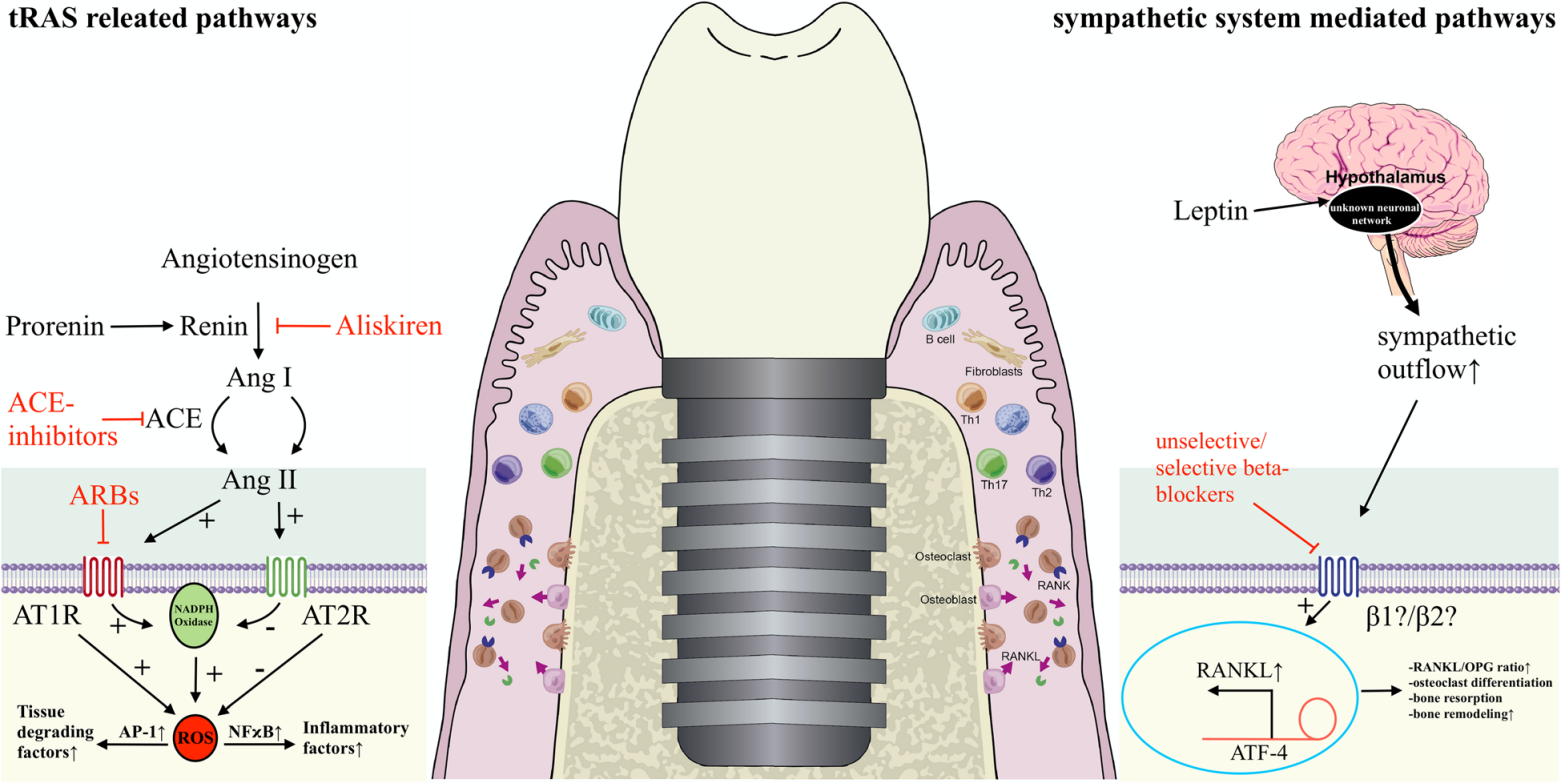
Illustration of the potential role of beta-blockers and RAS inhibitors in peri-implant bone remodeling processes. AP-1, activator protein 1; ARB, angiotensin II type 1 receptor blocker; RANKL, receptor activator of nuclear factor-kappa β ligand; OPG, osteoprotegerin; ATF-4, activating transcription factor 4; ROS, reactive oxygen species; NF-kβ, nuclear factor-kappa β; ACE, angiotensin-converting enzyme; Ang, angiotensin; AT1R, angiotensin II type 1 receptor; AT2R, angiotensin II type 2 receptor

Peri-implant bone remodeling processes contain inter alia a complex interaction between osteoblasts, osteoclasts, fibroblasts, immune cells, and mainly four factors, macrophage colony-stimulating factor (M-CSF), osteoprotegerin (OPG), receptor activator of nuclear factor-kappa β (RANK), and its ligand RANKL (Fig. 1) [25, 26]. Beta-adrenoreceptors are expressed on osteoblasts and are activated through leptin signaling via unknown hypothalamus neuronal networks and sympathetic nervous system activation, which leads to an increased receptor activator of nuclear factor-kappa β ligand (RANKL) release via activating transcription factor 4 (ATF-4) and consequently reduced bone formation [27–32]. The type of beta-adrenergic receptors on osteoblasts through which these effects are mediated remains controversial [29]. Further, possible mechanisms of the tissue renin-angiotensin system of the periodontal tissue cells are illustrated. RAS inhibitors may reduce peri-implant inflammation and tissue degradation through downregulating ATII-mediated pathways such as the activation of nuclear factor-kappa β (NF-kβ) and activator protein 1 (AP-1) via an increased intracellular concentration of reactive oxygen species (ROS) [33–35]. The effect of beta-blockers on bone is considered to be related to the role of the sympathetic nervous system: beta-2-adrenergic receptors were identified on osteoblasts and osteoclasts, and beta-adrenergic stimulation and inhibition can lead to anabolic and catabolic effects on bone [17, 19, 23]. Whereas robust evidence exists to suggest that beta-blockers may be positively associated with higher bone densities, there is limited evidence on RAS inhibitors and beta-blockers compared with each other in a clinical trial. A large nationwide case-control study showed a similar reduction in fracture risk between RAS inhibitors (7%) and beta-blockers (9%) [19].

Sufficient bone mineral density and healthy bone metabolism are important requirements for the osseointegration of dental implants. With the introduction of resonance frequency analysis in the 1990s, primary and secondary implant stability measurements became feasible [36–38]. Whereas primary implant stability depends on bone morphology and implant characteristics, secondary implant stability considers the physiological tissue response to the implant and subsequent bone remodeling processes, indicating successful osseointegration and implant success [39, 40]. The output of the implant stability measurements can be evaluated via the implant stability quotient (ISQ) and should be ≥ 60 to assume sufficient implant stability [41–43].

Currently, there is only one study to assess the impact of antihypertensive medication on implant survival [44]. The authors found higher survival rates in patients treated with antihypertensive medication compared to non-users. Of this cohort, containing 586 non-users and 142 antihypertensive drug users, the majority (54%) were taking RAS inhibitors, 18.9% beta-blockers, 5.4% thiazide diuretics, and 21.6% other drugs. However, they did not differentiate between the different antihypertensive subgroups regarding the survival rates of the implants.

Therefore, this study’s main aim was to investigate the impact of antihypertensive drug use on primary and secondary implant stability with a special focus on two frequently prescribed antihypertensive drug classes, beta-blockers and RAS inhibitors.

## Materials and methods

This study was conducted according to the Strengthening the Reporting of Observational Studies in Epidemiology (STROBE) guidelines [45]. The retrospective study was performed following the revised principles stated in the Declaration of Helsinki and the Good Clinical Practice Guidelines. This retrospective study is exempt from institutional review board approval by the State Medical Council of Baden-Würrtemberg, Germany. All patients gave their informed consent for inclusion before they participated in the study.

We carried out a retrospective cohort study based on a statistical analysis of anonymized data from a patient cohort treated at a tertiary outpatient clinic between 2015 and 2020. The inclusion criteria were as follows: (1) patients treated with at least one dental implant between 2015 and 2020, (2) age >18, (3) primary and secondary implant stability measured with radiofrequency analysis; (4) periodontally healthy subjects and no history of severe periodontitis. The exclusion criteria were as follows: (1) medication that could affect bone metabolism (antiresorptive drugs (bisphosphonates, selective estrogen modulators, denosumab), phenytoin, systemic glucocorticoids); (2) antihypertensive medication with drugs other than beta-blockers or RAS inhibitors; (3) required bone augmentation; (4) heavy smokers (>20 cigarettes per day); (5) disease which could affect bone metabolism (hyperthyroidism, rheumatoid arthritis, Paget’s disease, osteomalacia); (6) severe systemic diseases (American Society of Anesthesiology III or IV); (7) pregnant patients; (8) a handicap that would impair good oral hygiene. All consecutive patients treated between 2015 and 2020 and matching the predefined inclusion and exclusion criteria were included.

Patients were defined as antihypertensive drug users who reported taking beta-blockers (BB) and/or RAS inhibitors (ACE-inhibitors, ARBs, renin-inhibitors) at the preoperative clinical examination. Other antihypertensive drugs were not considered due to the small sample sizes. Based on this approach, four groups were built: patients with no antihypertensive medication (NAH group), patients with antihypertensive medication using beta-blockers (BB group), patients with antihypertensive medication using beta-blockers and RAS-inhibitors (Comb group), and finally, patients with antihypertensive medication using RAS-inhibitors (RAS group). Blood pressure data were not available for both the non-users and antihypertensive patients and could not be considered in our statistical analysis.

A standardized explanatory meeting was conducted before surgery, including questionaries and assessments of medical records, clinical examination, and radiographic diagnostics. Two board-certified craniomaxillofacial surgeons performed the surgeries and RFA measurements. Two implant systems were used for implant therapy: SLA® Straumann implant (STR) (Straumann Holding AG, Basel, Switzerland) and Inicell® Thommen implant (SPI) (Thommen Medical AG, Grenchen, Switzerland). RFA measurements were performed at implant insertion (timepoint: primary stability) and at implant exposure, approximately 120 days after implant insertion (timepoint: secondary stability). ISQ values were determined using Osstell Mentor™ (Integration Diagnostics Ltd., Goteborg, Sweden). The scale ranges from 1 to 100, whereas the “clinical range” with normally achievable values is 55–85. High stability means >70 ISQ, between 60 and 69 is considered medium stability, and < 60 ISQ is considered as low stability according to available evidence. Palatinal and vestibular measurements were averaged for statistical analyses.

### Data management and statistics

Descriptive statistics were performed to assess population characteristics. The odds ratio was calculated with its 95% confidence interval to compare antihypertensive drug users and non-users regarding the distribution of study variables. The mean is shown with standard deviation, whereas the median is shown with range. The Shapiro-Wilk normality test was applied. The Kruskal-Wallis test and the Mann-Whitney *U* test were used for nonparametric testing of study groups. We defined ISQ values at implant insertion (primary implant stability) and ISQ values at implant exposure (secondary implant stability) as primary outcomes to analyze the study variables’ potential effects on both time points. The relationship between implant stability and study groups (NAH vs. BB vs. Comb vs. RAS) was assessed via a multivariable linear mixed regression model. The model included the following confounders: age, sex (women vs. men), jaw (mandibular vs. maxillary), teeth region (front vs. premolar vs. molar), bone status, implant insertion technique (immediate insertion vs. internal sinus elevation vs. one-step/two-step insertion after sinus elevation), implant system (STR vs. SPI), and healing time. Healing time was defined as the time between implant insertion (primary stability) and implant exposure (secondary stability) in weeks. Before fitting a multivariable model, each of these variables was assessed with a single linear regression model. Consequently, a multivariable linear mixed regression model was applied, and a regression coefficient, with its 95% confidence interval, was calculated to examine the relationship between the primary outcomes and the study groups. Primary implant stability was included in the multivariable linear mixed regression model of the second time point. A two-sided *p*-value < 0.05 was considered to be statistically significant. Statistical analysis was performed using Stata Statistical Software Release 15 (StataCorp. 2011, College Station, TX, USA).

## Results

### Population and group characteristics

Overall, 196 patients with a total of 377 implants were enrolled in this study (96 (49.0%) females and 100 (51.02%) males, with a median age of 65 years (range 26–93)); 179 (47.5%) dental implants were placed in females whereas 198 (52.52%) were implanted in males. A total of 331/377 (87.8%) implants were placed in NAH, whereas 46/377 (12.2%) were placed in AH. Among the implants inserted in the AH group, 13 (28.26%) were inserted in BB users, 11 (23.9%) in Comb users, and 22 (47.8%) in RAS users. Further, three of the BB group implants were inserted in unselective beta-blocker users, whereas no implant in the Comb group was inserted in unselective beta-blocker users. The RAS group included 13 (59.1%) ACE users and 9 (40.9%) ARB users. The ACE group contained ramipril (10/13, 76.9%) and lisinopril (3/13, 23.1%). Candesartan (7/9, 77.8%) was the most often used ARB, followed by irbesartan (2/9, 22.2%). Renin inhibitor users were not present in our cohort. Our implant characteristics and therapeutic approaches were equally distributed among antihypertensive drug users and non-users (Table 1). Age was the only study variable that revealed significant differences between study groups: patients ≥70 years old were more likely to take antihypertensive drugs than their younger comparators (< 70-year-old) (OR 2.30; 95% CI 1.17–4.64; *p*=0.009).

**Table 1:** Comparison of antihypertensive drug users and non-users by implants (*n*=377)

Variable	All *n*	Antihypertensive drug		OR (95% CI)	*P*
		Users *n* (%)	Non-users *n* (%)		
Gender					
Female	179	23 (50.0)	156 (47.1)	1	
Male	198	23 (50.0)	175 (52.9)	0.89 (0.46–1.74)	ns
Age					
<70	207	17 (37.0)	190 (57.4)	1	
≥70	170	29 (63.0)	141 (42.6)	2.30 (1.17–4.64)	0.01*
Implant system					
STR	289	30 (65.2)	259 (78.3)	1	
SPI	88	16 (34.8)	72 (21.8)	1.92 (0.92–3.86)	ns
Implant diameter					
4.1 mm	187	20 (43.5)	167 (50.5)	1	
< 4.1 mm	63	3 (6.5)	60 (18.1)	0.42 (0.08–1.49)	ns
> 4.1 mm	127	23 (50.0)	104 (31.4)	1.85 (0.92–3.73)	ns
Implant length					
<11 mm	169	25 (54.4)	144 (43.5)	1	
≥11 mm	208	21 (45.7)	187 (56.5)	0.65 (0.33–1.26)	ns
Localization					
Maxillary	225	23 (50.0)	202 (61.0)	1	
Mandibular	152	23 (50.0)	129 (39.0)	1.57 (0.80–3.05)	ns
Healing time					
< 14 weeks	162	20 (43.5)	142 (42.9)	1	
> 14 weeks	215	26 (56.5)	189 (57.1)	0.98 (0.50–1.93)	ns
Bone situation/implant technique					
Fully ossified situation	305	38 (82.6)	267 (80.7)	1	
Immediate insertion	41	3 (6.5)	38 (11.5)	0.56 (0.11–1.89)	ns
Internal sinus elevation (immediate insertion)^a^	9	0 (0.0)	9 (2.72)	-	-
One-step/two-step insertion after internal sinus elevation	22	5 (10.87)	17 (5.14)	2.07 (0.56–6.27)	ns

*STR* Straumann implants, *SPI* Thommen implants, *p p*-value, *OR* odds ratio, *95% CI* 95% confidence interval, *indicate significance, *p p*-value, *ns* not significant

^a^Calculating the odds ratio with its confidence interval for the zero-cell counts via mathematical correction (Haldane-Anscombe correction) was not necessary, as this group did not contain any antihypertensive drug users

### Implant and bone characteristics

**Fig. 2:**
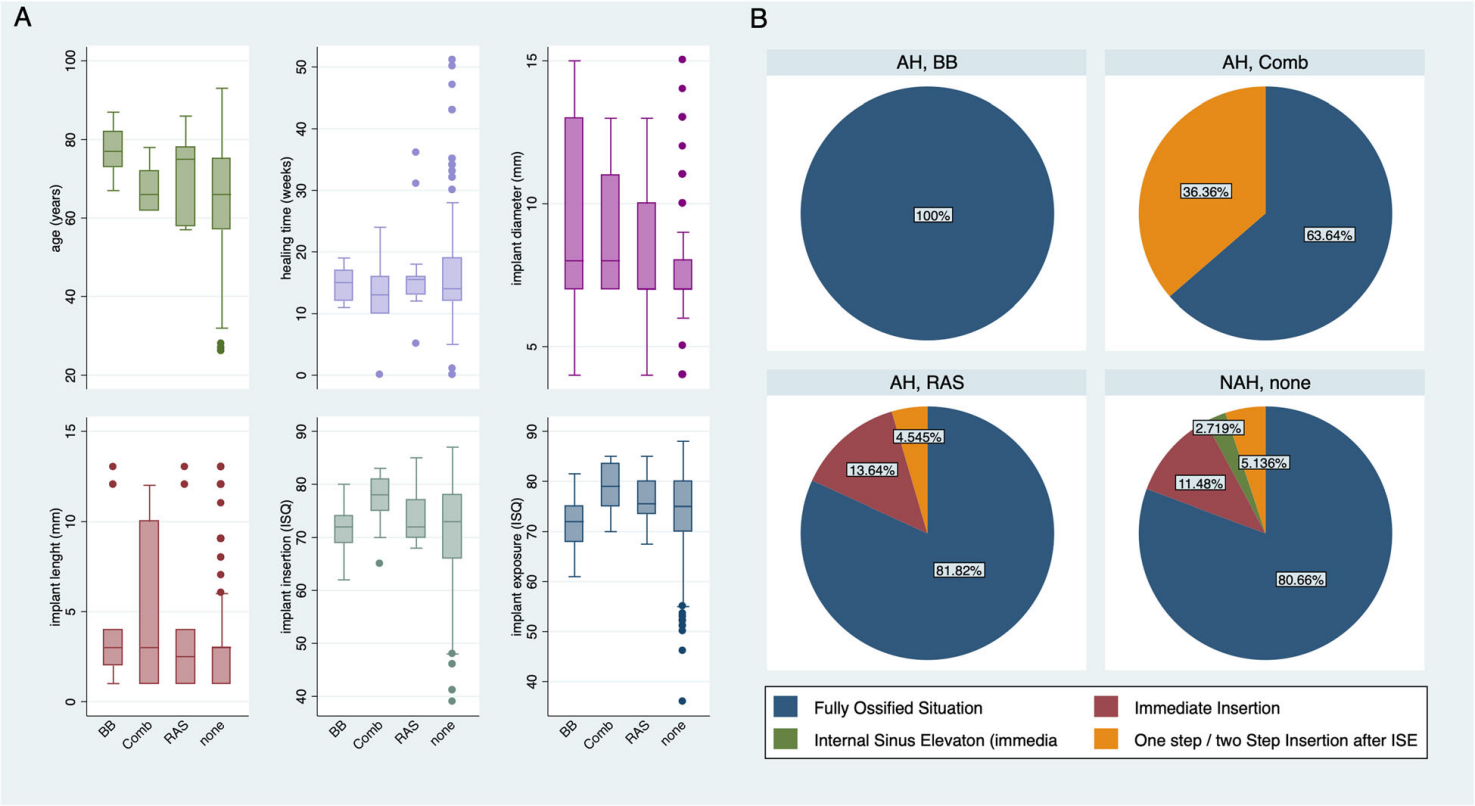
Distribution of study variables in the study groups via box plots (median and interquartile range) (**a**) and pie charts showing the implant insertion technique’s distribution among the study groups (**b**). BB, beta-blocker group; Comb, combined beta-blocker and RAS-inhibitor group; RAS, RAS inhibitor group; ISE, internal sinus elevation; ISQ, implant stability quotient

The number of implants per case ranged from 1 to 10 (1.92 ± 1.3). A higher number of maxillary implants (225/377, 59.7%) were inserted compared to mandibular (152/377, 40.3%) implants. The predominant teeth region was the molar region (139/377, 36.9%), followed by the premolar region (35.8%), and the front teeth region (103/377, 27.32%). From all inserted implants, 289 (76.7%) were STR and 88 (23.3%) were SPI. The mean healing time, defined as the time between implant insertion and implant exposure, was 117.1 ± 56.6 days. Implant diameters and length ranged from 3.3 to 6mm and 4 to 14.5mm, respectively. A total of 305/377 implants were inserted in fully ossified bone situations, 41/377 (10.9%) were inserted immediately after teeth extraction (immediate insertion), 9/377 (2.4%) were inserted after internal sinus elevation with immediate insertion, and finally, 22/377 (5.84%) were inserted in a one-step/two-step insertion procedure after sinus elevation. No significant differences between the study groups were found in the pairwise analysis. Implant insertion technique and the bone situation at implant insertion were not similarly distributed between the different antihypertensive drug groups as assessed with descriptive statistics (Fig. 2). Therefore, we included this parameter as a potential confounder in our regression model.

### Study outcomes at implant insertion

**Fig. 3:**
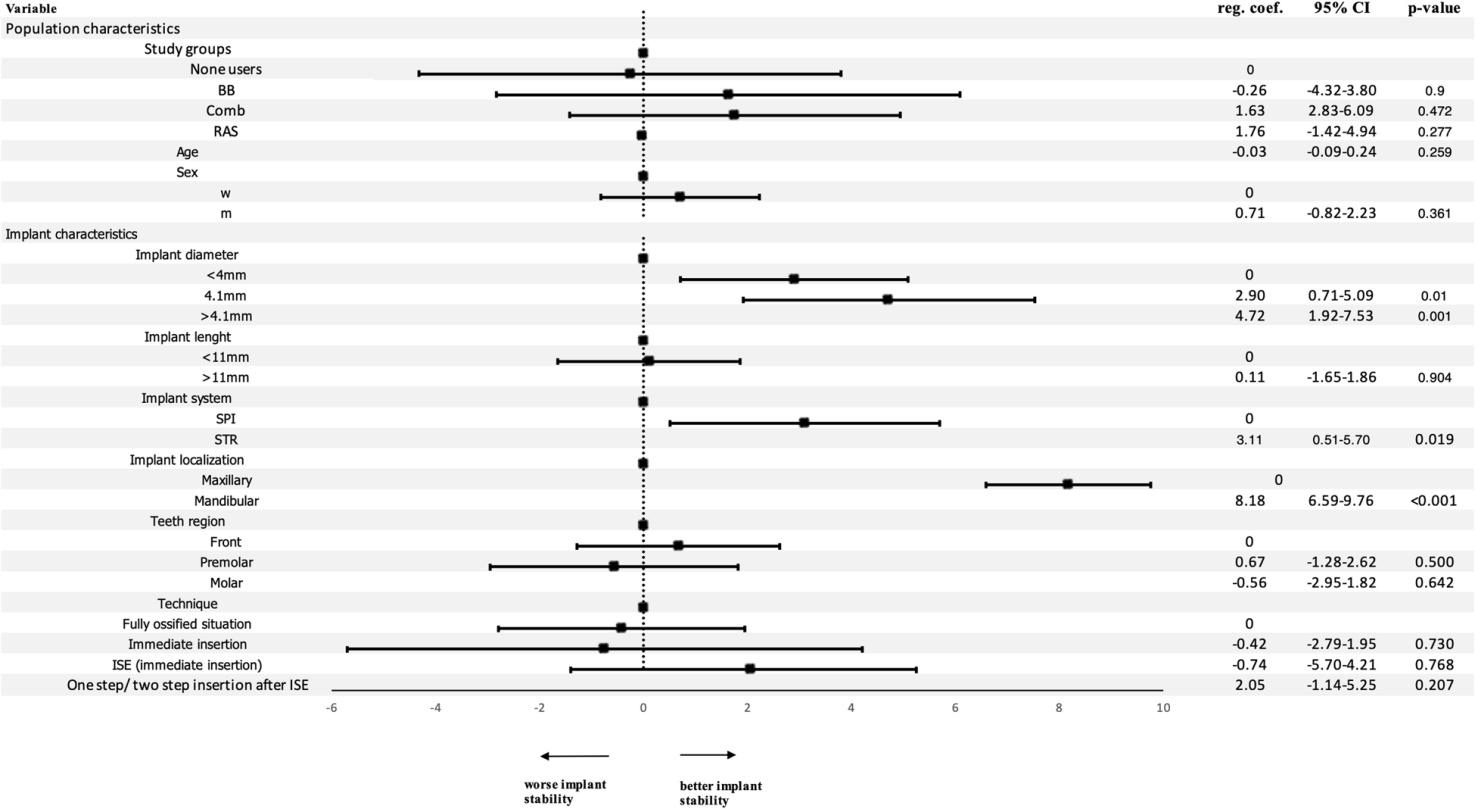
Regression model of the implant stability quotient (ISQ) at implant insertion. BB, beta-blocker group; Comb, combined beta-blocker and RAS-inhibitor group; RAS, RAS inhibitor group; m, male; f, female; ISE, internal sinus elevation; STR, Straumann implants; SPI, Thommen implants; reg. coeff., regression coefficient; 95% CI 95% confidence interval

ISQ values at insertion were 71.8 ± 8.7 (95% CI 70.83*–*72.70) for NAH and 74.1 ± 5.6 (95% CI 72.42*–*75.71) for AH, respectively. ISQ values for the AH subgroups were 71.7 ± 5.4 (95% CI 68.42*–*74.97) for BB, 77.0 ± 5.5 (95% CI 73.32*–*80.68) for Comb, and finally, 74.0 ± 5.2 (95% CI 71.70*–*76.30) for the RAS inhibitor group. No significant association was found for the antihypertensive subgroups and the ISQ at implant insertion value after adjusting for the confounding factors, age, sex, implant characteristics (implant diameter, length, system, localization, insertion technique, or teeth region) (Fig. 3). However, an implant diameter of 4.1 mm (reg. coeff. 2.90, 95% CI 0.71*–*5.09; *p*=0.01) and > 4.1mm (reg. coeff. 4.72, 95% CI 1.92*–*7.53; *p*<0.001) showed higher implant stability values than an implant diameter < 4mm. Straumann implants showed higher ISQ values at implant insertion (reg. coeff. 3.11, 95% CI 0.51*–*5.70; *p*=0.019) compared to Thommen implants. Further, mandibular implants (reg. coeff. 8.18, 95% CI 6.59*–*9.76; *p*<0.001) were associated with higher ISQ values compared to maxillary implants. Interestingly, implant insertion technique and the bone situation at implant insertion (fully ossified bone vs. immediate insertion vs. internal sinus elevation (ISE) with immediate insertion vs. one-step/two-step insertion after ISE), as well as teeth region (front vs. premolar vs. molar) did not reveal significant differences in our regression model.

### Study outcomes at implant exposure

**Fig. 4:**
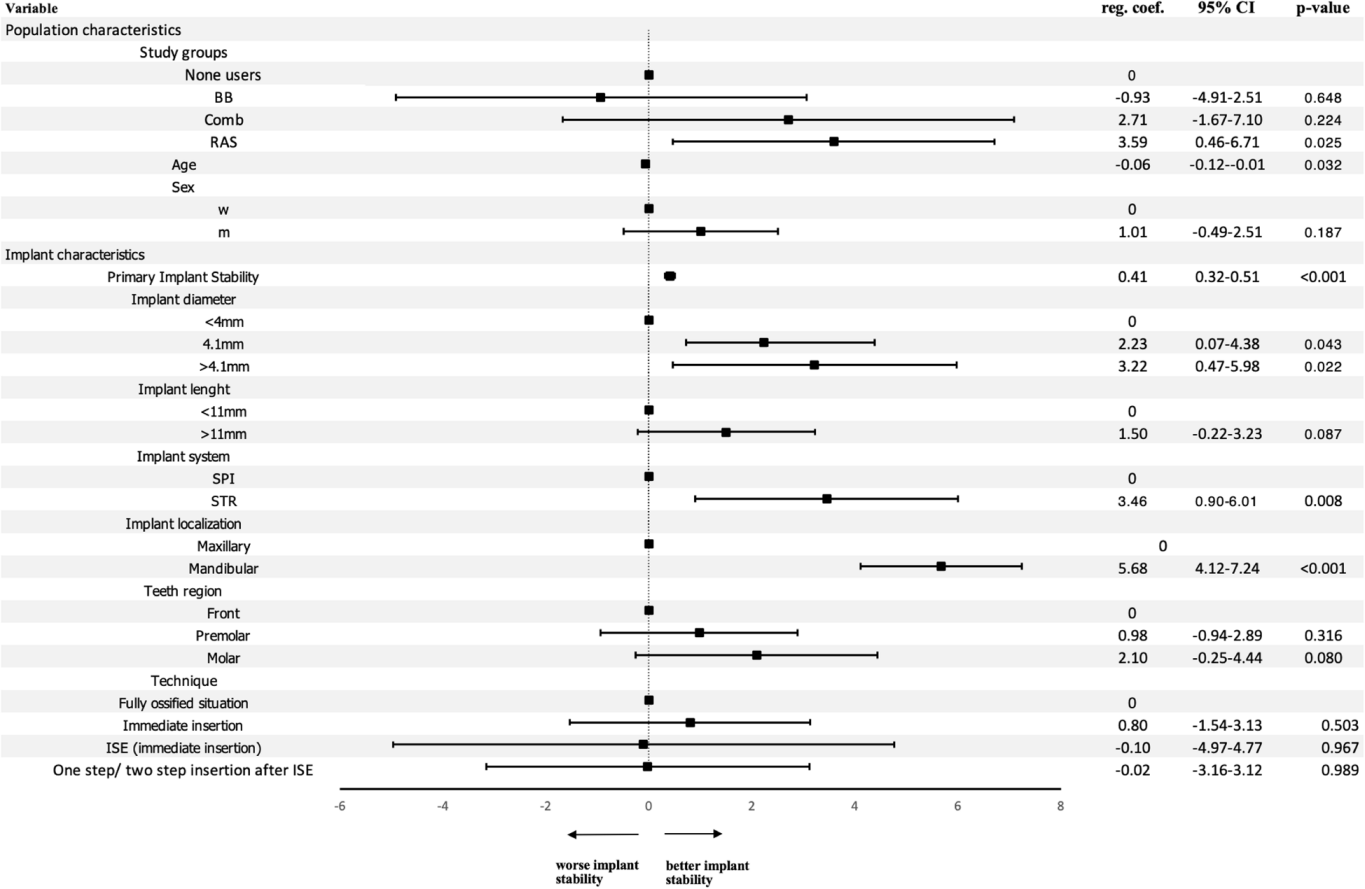
Regression model of the implant stability quotient (ISQ) at implant exposure. BB, beta-blocker group; Comb, combined beta-blocker and RAS-inhibitor group; RAS, RAS inhibitor group; m, male; f, female; ISE, internal sinus elevation; STR, Straumann implants; SPI, Thommen implants; reg. coeff., regression coefficient; 95% CI, 95% confidence interval

ISQ values at exposure were 73.7 ± 8.1 (95% CI 72.77*–*74.53) for NAH and 75.7 ± 5.9 (95% CI 73.99*–*77.48) for AH, respectively. ISQ values for the AH subgroups were 72.00 ± 6.4 (95% CI 68.11*–*75.89) for BB, 78.36 ± 5.1 (95% CI 74.97*–*81.76) for Comb, and finally, 76.64 ± 5.0 (95% CI 74.43*–*78.84) for the RAS inhibitor group. A significant association between antihypertensive drug subgroups and implant stability at implant exposure was found for the RAS inhibitor group (reg. coeff. 3.59, 95% CI 0.46*–*6.71; *p*<0.05), whereas the combined beta-blocker/RAS inhibitor group (reg. coeff. 2.71, 95% CI *−* 1.67*–*7.10; *p*=0.224) and the beta-blocker group (reg. coeff. *−*0.93, 95% CI *−*4.91*–*2.51; *p*=0.648) failed to reveal statistical significance (Fig. 4). Equivalent to the implant insertion findings, implant diameter, system, and localization were significantly associated with implant stability. Additionally, a significant correlation between ISQ values at implant insertion and ISQ values at implant exposure were found in our regression model for the dependent variable secondary implant stability (reg. coeff. 0.41, 95% CI 0.32*–*0.51; *p*<0.001). Moreover, healing time (in weeks) between implant insertion and exposure showed no statistical differences for implant stability at implant exposure when adjusted for the other study variables (reg. coeff. 0.07, 95% CI −0.02–0.16; *p*=0.11) and was further equally distributed among the subgroups as illustrated in Fig. 2.

## Discussion

We provided first-time clinical data regarding the impact of, and the difference between, frequently used and relevant antihypertensive drug groups, and implant stability assessed via radiofrequency analysis and expressed with the implant stability quotient (ISQ).

Outcomes reveal that RAS inhibitors were significantly associated with higher implant stability values at implant exposure than non-users. In contrast, a comparison of beta-blockers versus non-users did not indicate a significant association in our regression model. These findings are of high interest for dental implant surgeons and hypertensive patients before implant therapy, as this patient cohort might benefit from RAS inhibition.

Sufficient implant stability is a measure for successful osseointegration and, consequently, implant success. Our work partly confirms previous findings suggesting antihypertensive medication having beneficial effects on implant stability [44]. Yet, the protective effect of beta-blockers on bone-related parameters and dental implant success could not be observed in the present study [46, 47]. There was a tendency in patients taking both beta-blockers and RAS inhibitors to achieve higher implant stability at implant insertion and exposure. However, these findings failed to reach significance. We assume there will be different results in future prospective studies when more patients are included in this group. In contrast, in the group solely taking beta-blockers, there were no clear indications that the users achieved higher implant stability values. In a recent systematic review that assessed medication-related dental implant failures, only one study could be found investigating the impact of antihypertensive medication on implant survival [48]. Consequently, no meta-analysis could be performed because of the limited available evidence. This study revealed higher implant survival rates for patients receiving antihypertensive drug medication [44]. Interestingly, most of the patients included in this study (54%) were taking RAS-inhibitors, whereas only 18.0% were taking beta-blockers, and 5.4% thiazide diuretics, which indicates that RAS-inhibitor medication group could have led to this higher survival rates. However, no subgroup analysis was performed, limiting definitive conclusions on this topic. Another recent retrospective cohort study found higher peri-implantitis rates and higher deep-pocket rates in patients prescribed with antihypertensive drugs [49]. Also here, the authors did not discriminate between the different antihypertensive drug classes, and the study further focused on other outcome measures, thus, limiting the comparability with the present study.

It must be considered that we did not differentiate between selective and unselective beta-blockers in our statistical analyses because of the relatively small study population. In our study cohort, 3/13 implants (23.1%) in the beta-blockers only group were inserted in patients taking unselective beta-blockers, whereas no implant in the combined beta-blockers and RAS-inhibitor group was inserted in unselective beta-blocker users. There is evidence that selective beta-blockers might be positively associated with higher bone mineral densities and other implant success relevant parameters, whereas this effect was not confirmed for unselective beta-blockers, such as propranolol [50–52]. Therefore, future studies might come to different conclusions if selective beta-blockers are investigated solely.

Current research provides robust data on RAS-inhibitors and their positive effects on bone metabolism and dental implant success [35, 44, 53]. We recently conducted a systematic review and network meta-analysis in which we summarized the current preclinical evidence regarding the tissue renin-angiotensin system, its inhibition, and periodontal bone remodeling outcomes [13]. Our results showed significant positive effects of RAS inhibition on tissue inflammation and bone remodeling markers. However, the findings of the present study are indicative. Hence, we cannot conclude that taking RAS-inhibitors is generally associated with higher implant stability, as we only found a correlation between RAS inhibitor usage and higher implant stability values at implant exposure. However, for the first time, we have provided real-world data revealing RAS inhibitors having protective effects on primary and secondary implant stability following dental implant surgery. Secondary implant stability is dependent on bone remodeling processes and, therefore, biological responses of the bone, whereas primary implant stability highly depends on bone-related factors (quantity and quality) and implant-related factors (diameter, length, and surface characteristics) [54, 55]. In summary, the present work allows us to conclude that RAS inhibitors could positively affect periodontal bone remodeling processes, as shown in previous preclinical studies [12, 16, 56].

The present study is associated with strengths and limitations. To our best knowledge, this is the first study considering primary and secondary implant stability to evaluate the impact of antihypertensive medication on dental implant success. One further strength of this study is that we used appropriate statistical approaches to adjust for relevant potential confounders that could affect the outcome measures. As expected, antihypertensive drug users were older than non-users in our study. It is well-known that age-dependent changes affect the bone structure, mechanical behavior, and bone metabolism in the periodontal tissue [57]. Interestingly, we found significantly, though low expressed, decreasing secondary implant stability values with increasing age, as seen in our regression analysis. In contrast, we found no associations between age and primary implant stability values, confirming that primary implant stability might be less dependent on biological factors. Furthermore, here we provide the first data stratifying between different antihypertensive drug classes when evaluating implant stability. Consequently, this is currently the largest dataset on the impact of antihypertensive drug use and implant stability.

Nevertheless, the retrospective character of our study is associated with some intrinsic limitations, such as selective bias due to the inclusion of a selected cohort, which probably cannot be generalized for all antihypertensive drug users, lack of randomization, and matched control comparisons. However, we considered confounding variables, such as age and sex, which are often used to match a control group, in our regression model, allowing for a proper comparison between the study groups. Furthermore, the present odds ratio comprised a similar distribution of study variables between the antihypertensive user and non-user groups. Limited by the retrospective design and the small study size of the antihypertensive group, we could not adjust for other potentially relevant confounding variables, such as different disease status or medications, which might be affecting our primary outcomes. However, we used strict inclusion and exclusion criteria to obtain a homogenous patient cohort with diseases and medications that likely do not affect bone metabolism. Notably, our cohort included only patients with healthy periodontal status and without a history of severe periodontitis. These data were provided in the medical examination and were accurately documented. This counteracted some bias that may be possible in retrospective designs, as patients with healed periodontitis, even though a specific time frame after periodontal therapy has been exceeded, have been shown to have a lower implant success rated compared to patients who had no history of periodontal diseases [58]. Further, antihypertensive drug users and non-users had ISQ values >70, which is currently considered “high implant stability”. Therefore, the clinical relevance of the difference between the groups remains theoretical. The proof of clinically relevant loss of implant stability would require clinical settings with many patients who were affected by low implant stability and examination of the loss of implant stability during long follow-ups, which was not the case in our study. However, our main aim was to prove if there is a significant association between ISQ values and the study groups, regardless of the obtained ISQ levels, which we could confirm for ISQ values at implant exposure. Future prospective studies could include more extended follow-up examinations with multiple implant stability measurements and more outcomes, such as implant failure rates or radiological examinations, to evaluate a clinically relevant loss of success between the groups. Additionally, the present results can further be used in future mathematical prediction models to predict implant stability values based on different confounding variables. We did not consider blood pressure differences between the patients as this data is not routinely available in retrospective obtained data from dental implant patients. Manrique et al. showed in a model of spontaneously hypertensive rats that hypertension affected bone healing and decreased bone thickness [59]. Nevertheless, there is currently no evidence from clinical studies confirming an association between high blood pressure and decreased osseointegration of dental implants. Blood pressure could be considered in future studies to evaluate the impact on implant success. Notably, we did not evaluate the duration of the drug use in the cohort, as these data are not routinely evaluated in clinics. However, as our AH group contained mainly old patients and these patient group often remain on blood pressure agents for a long time, we suggest that all patients in this group consisted of long-term AH drug users leading to appropriate comparability within this group comparing to cohorts with young patients, where the drug intake duration may show high variabilities. Additionally, we did not differentiate between selective and unselective beta-blockers, which are reported to have a different impact on bone metabolism and implant success, due to the small study population. Hence, we recommend future prospective studies to differentiate between beta-blocker groups. Moreover, after stratification by antihypertensive drugs, the group power of the beta-blocker, combined beta-blocker, and RAS-inhibitor group was relatively small.

## Conclusions

RAS-inhibitor usage is associated with enhanced implant stability at implant exposure following implant therapy than in non-users. The protective effect of beta-blockers on implant stability could not be confirmed in the present work. Future large prospective randomized controlled trials are warranted to prove these indicative findings.

## Data Availability

Datasets are available from the corresponding author on reasonable request. The raw data and all related documents supporting the conclusions of this manuscript will be made available by the authors, without undue reservation, to any qualified researcher.

## References

[1] Mills KT, Stefanescu A, He J (2020). The global epidemiology of hypertension. Nat Rev Nephrol.

[2] Bromfield S, Muntner P (2013). High blood pressure: the leading global burden of disease risk factor and the need for worldwide prevention programs. Curr Hypertens Rep.

[3] Lim SS, Vos T, Flaxman AD, Danaei G, Shibuya K, Adair-Rohani H (2012). A comparative risk assessment of burden of disease and injury attributable to 67 risk factors and risk factor clusters in 21 regions, 1990–2010: a systematic analysis for the Global Burden of Disease Study 2010. Lancet.

[4] Jarari N, Rao N, Peela JR, Ellafi KA, Shakila S, Said AR (2015). A review on prescribing patterns of antihypertensive drugs. Clin Hypertens.

[5] James PA, Oparil S, Carter BL, Cushman WC, Dennison-Himmelfarb C, Handler J (2014). 2014 Evidence-based guideline for the management of high blood pressure in adults: report from the panel members appointed to the Eighth Joint National Committee (JNC 8). JAMA.

[6] Gu Q, Paulose-Ram R, Dillon C, Burt V (2006). Antihypertensive medication use among US adults with hypertension. Circulation.

[7] Ma J, Lee K-V, Stafford RS (2006). Changes in antihypertensive prescribing during US outpatient visits for uncomplicated hypertension between 1993 and 2004. Hypertension.

[8] Ong KL, Cheung BMY, Man YB, Lau CP, Lam KSL (2007). Prevalence, awareness, treatment, and control of hypertension among United States adults 1999–2004. Hypertension.

[9] Kassebaum NJ, Bernabé E, Dahiya M, Bhandari B, Murray CJL, Marcenes W (2014). Global burden of severe tooth loss: a systematic review and meta-analysis. J Dent Res.

[10] Muñoz Aguilera E, Suvan J, Buti J, Czesnikiewicz-Guzik M, Barbosa Ribeiro A, Orlandi M (2020). Periodontitis is associated with hypertension: a systematic review and meta-analysis. Cardiovasc Res.

[11] Paizan M, Vilela-Martin J (2014). Is there an association between periodontitis and hypertension?. CCR.

[12] Santos CF, Morandini AC, Dionisio TJ, Faria FA, Lima MC, Figueiredo CM (2015). Functional local renin-angiotensin system in human and rat periodontal tissue. PLoS One.

[13] Saravi B, Lang G, Ülkümen S, Burchard T, Weihrauch V, Patzelt S (2020). The tissue renin-angiotensin system (tRAS) and the impact of its inhibition on inflammation and bone loss in the periodontal tissue. Eur Cell Mater.

[14] Araujo AA, Moura LM, Brito GAC, Aragao KS, Araujo LS, Souza TO (2013). Effect of telmisartan on levels of IL-1, TNF-alpha, down-regulated COX-2, MMP-2, MMP-9 and RANKL/RANK in an experimental periodontitis model. J Clin Periodontol.

[15] Araujo AA, Lopes de Souza G, Souza TO, de Castro Brito GA, Saboia Aragao K, Xavier de Medeiros CA (2013). Olmesartan decreases IL-1beta and TNF-alpha levels; downregulates MMP-2, MMP-9, COX-2, and RANKL; and upregulates OPG in experimental periodontitis. Naunyn Schmiedebergs Arch Pharmacol.

[16] Gabriele LG, Morandini AC, Dionisio TJ, Santos CF (2017). Angiotensin II type 1 receptor knockdown impairs interleukin-1beta-induced cytokines in human periodontal fibroblasts. J Periodontol.

[17] Ilić K, Obradović N, Vujasinović-Stupar N (2013). The relationship among hypertension, antihypertensive medications, and osteoporosis: a narrative review. Calcif Tissue Int.

[18] Perez-Castrillon J, Justo I, Sanz-Cantalapiedra A, Pueyo C, Hernandez G, Duenas A (2005). Effect of the antihypertensive treatment on the bone mineral density and osteoporotic fracture. CHYR.

[19] Rejnmark L, Vestergaard P, Mosekilde L (2006). Treatment with beta-blockers, ACE inhibitors, and calcium-channel blockers is associated with a reduced fracture risk: a nationwide case–control study. J Hypertens.

[20] Lynn H, Kwok T, Wong SYS, Woo J, Leung PC (2006). Angiotensin converting enzyme inhibitor use is associated with higher bone mineral density in elderly Chinese. Bone.

[21] Ağaçayak KS, Güven S, Koparal M, Güneş N, Atalay Y, Atılgan S (2014). Long-term effects of antihypertensive medications on bone mineral density in men older than 55 years. Clin Interv Aging.

[22] Hijazi N, Alourfi Z (2020). Association between hypertension, antihypertensive drugs, and osteoporosis in postmenopausal Syrian women: a cross-sectional study. Adv Med.

[23] Pasco JA, Henry MJ, Sanders KM, Kotowicz MA, Seeman E, Nicholson GC (2003). β-Adrenergic blockers reduce the risk of fracture partly by increasing bone mineral density: Geelong Osteoporosis Study. J Bone Miner Res.

[24] Schlienger RG (2004). Use of β-blockers and risk of fractures. JAMA.

[25] Kumar G, Roger P-M (2019). From crosstalk between immune and bone cells to bone erosion in Infection. IJMS.

[26] Ono T, Hayashi M, Sasaki F, Nakashima T (2020). RANKL biology: bone metabolism, the immune system, and beyond. Inflamm Regener.

[27] Ducy P, Amling M, Takeda S, Priemel M, Schilling AF, Beil FT (2000). Leptin inhibits bone formation through a hypothalamic relay. Cell.

[28] Elefteriou F, Ahn JD, Takeda S, Starbuck M, Yang X, Liu X (2005). Leptin regulation of bone resorption by the sympathetic nervous system and CART. Nature.

[29] Lafage-Proust MH (2019). Beta-blockers for osteoporosis: the sequel. Kidney Int.

[30] Kajimura D, Hinoi E, Ferron M, Kode A, Riley KJ, Zhou B (2011). Genetic determination of the cellular basis of the sympathetic regulation of bone mass accrual. J Exp Med.

[31] Flier JS (2002). Is brain sympathetic to bone?. Nature.

[32] Takeda S, Elefteriou F, Levasseur R, Liu X, Zhao L, Parker KL (2002). Leptin regulates bone formation via the sympathetic nervous system. Cell.

[33] Villar-Cheda B, Costa-Besada MA, Valenzuela R, Perez-Costas E, Melendez-Ferro M, Labandeira-Garcia JL (2017). The intracellular angiotensin system buffers deleterious effects of the extracellular paracrine system. Cell Death Dis.

[34] Nakamura T, Hasegawa-Nakamura K, Sakoda K, Matsuyama T, Noguchi K (2011). Involvement of angiotensin II type 1 receptors in interleukin-1beta-induced interleukin-6 production in human gingival fibroblasts. Eur J Oral Sci.

[35] Zhao J, Yang H, Chen B, Zhang R (2019). The skeletal renin-angiotensin system: a potential therapeutic target for the treatment of osteoarticular diseases. Int Immunopharmacol.

[36] Meredith N, Alleyne D, Cawley P (1996). Quantitative determination of the stability of the implant-tissue interface using resonance frequency analysis. Clin Oral Implants Res.

[37] Meredith N (1998). Assessment of implant stability as a prognostic determinant. Int J Prosthodont.

[38] Javed F, Romanos GE (2010). The role of primary stability for successful immediate loading of dental implants. A literature review. J Dent.

[39] Mathieu V, Vayron R, Richard G, Lambert G, Naili S, Meningaud J-P (2014). Biomechanical determinants of the stability of dental implants: influence of the bone–implant interface properties. J Biomech.

[40] Vollmer A, Saravi B, Lang G, Adolphs N, Hazard D, Giers V (2020). Factors influencing primary and secondary implant stability—a retrospective cohort study with 582 implants in 272 patients. Appl Sci.

[41] Sennerby L, Meredith N (2008). Implant stability measurements using resonance frequency analysis: biological and biomechanical aspects and clinical implications. Periodontol 2000.

[42] Trisi P, Carlesi T, Colagiovanni M, Perfetti G (2010). Implant stability quotient (ISQ) vs direct in vitro measurement of primary stability (micromotion): effect of bone density and insertion torque. J Osteol Biomat.

[43] Pagliani L, Sennerby L, Petersson A, Verrocchi D, Volpe S, Andersson P (2013). The relationship between resonance frequency analysis (RFA) and lateral displacement of dental implants: an *in vitro* study. J Oral Rehabil.

[44] Wu X, Al-Abedalla K, Eimar H, Arekunnath Madathil S, Abi-Nader S, Daniel NG (2016). Antihypertensive medications and the survival rate of osseointegrated dental implants: a cohort study: antihypertensive drugs and dental implants. Clin Implant Dent Relat Res.

[45] von Elm E, Altman DG, Egger M, Pocock SJ, Gøtzsche PC, Vandenbroucke JP (2007). The strengthening the Reporting of Observational Studies in Epidemiology (STROBE) statement: guidelines for reporting observational studies. Prev Med.

[46] Luis Miguel SA, Pedro M, Sola-Martín C, José G-S, Juan L-Q (2017). Osseointegrated implants and beta-blockers drugs: is there any relationship?. Clin Oral Impl Res.

[47] Al-Subaie AE, Laurenti M, Abdallah M-N, Tamimi I, Yaghoubi F, Eimar H (2016). Propranolol enhances bone healing and implant osseointegration in rats tibiae. J Clin Periodontol.

[48] Chappuis V, Avila-Ortiz G, Araújo MG, Monje A (2018). Medication-related dental implant failure: Systematic review and meta-analysis. Clin Oral Impl Res.

[49] Seki K, Hasuike A, Iwano Y, Hagiwara Y (2020). Influence of antihypertensive medications on the clinical parameters of anodized dental implants: a retrospective cohort study. Int J Implant Dent.

[50] Yang S, Nguyen ND, Center JR, Eisman JA, Nguyen TV (2011). Association between beta-blocker use and fracture risk: the Dubbo Osteoporosis Epidemiology Study. Bone.

[51] Song HJ, Lee J, Kim Y-J, Jung S-Y, Kim HJ, Choi N-K (2012). β1 selectivity of β-blockers and reduced risk of fractures in elderly hypertension patients. Bone.

[52] Reid IR, Lucas J, Wattie D, Horne A, Bolland M, Gamble GD (2005). Effects of a β-blocker on bone turnover in normal postmenopausal women: a randomized controlled trial. J Clin Endocrinol Metab.

[53] Gebru Y, Diao T-Y, Pan H, Mukwaya E, Zhang Y (2013). Potential of RAS inhibition to improve metabolic bone disorders. Biomed Res Int.

[54] Brunski JB (1992). Biomechanical factors affecting the bone-dental implant interface. Clin Mater.

[55] Sennerby L, Roos J (1998). Surgical determinants of clinical success of osseointegrated oral implants: a review of the literature. Int J Prosthodont.

[56] Mulinari-Santos G, Santos JSD, Palin LP, Silva ACE d, Antoniali C, Faverani LP (2019). Losartan improves alveolar bone dynamics in normotensive rats but not in hypertensive rats. J Appl Oral Sci.

[57] Boskey AL, Coleman R (2010). Aging and bone. J Dent Res.

[58] Ramanauskaite A, Baseviciene N, Wang H-L, Tözüm TF. Effect of history of periodontitis on implant success: meta-analysis and systematic review. Implant Dent. 2014;23:687–96. 10.1097/ID.0000000000000156 25343317

[59] Manrique N, Pereira CCS, Luvizuto ER, Sánchez MDPR, Okamoto T, Okamoto R (2015). Hypertension modifies OPG, RANK, and RANKL expression during the dental socket bone healing process in spontaneously hypertensive rats. Clin Oral Invest.

